# Prediction of Pharmacokinetic Parameters Using a Genetic Algorithm Combined with an Artificial Neural Network for a Series of Alkaloid Drugs

**DOI:** 10.3797/scipharm.1306-10

**Published:** 2013-09-22

**Authors:** Majid Zandkarimi, Mohammad Shafiei, Farzin Hadizadeh, Mohammad Ali Darbandi, Kaveh Tabrizian

**Affiliations:** 1 Department of Pharmaceutical Sciences, School of Pharmacy, Zabol University of Medical Sciences, Zabol, Iran.; 2 Department of Medicinal Chemistry, School of Pharmacy, Mashad University of Medical Sciences, Mashad, Iran.; 3 Department of Pharmacology and Toxicology, School of Pharmacy, Zabol University of Medical Sciences, Zabol, Iran.

**Keywords:** Genetic Algorithm, Artificial Neural Network, Structural Descriptors, Alkaloid Drugs, Pharmacokinetic parameters

## Abstract

An important goal for drug development within the pharmaceutical industry is the application of simple methods to determine human pharmacokinetic parameters. Effective computing tools are able to increase scientists’ ability to make precise selections of chemical compounds in accordance with desired pharmacokinetic and safety profiles. This work presents a method for making predictions of the clearance, plasma protein binding, and volume of distribution for alkaloid drugs. The tools used in this method were genetic algorithms (GAs) combined with artificial neural networks (ANNs) and these were applied to select the most relevant molecular descriptors and to develop quantitative structure-pharmacokinetic relationship (QSPkR) models. Results showed that three-dimensional structural descriptors had more influence on QSPkR models. The models developed in this study were able to predict systemic clearance, volume of distribution, and plasma protein binding with normalized root mean square error (NRMSE) values of 0.151, 0.263, and 0.423, respectively. These results demonstrate an acceptable level of efficiency of the developed models for the prediction of pharmacokinetic parameters.

## Introduction

**Fig. 1:**
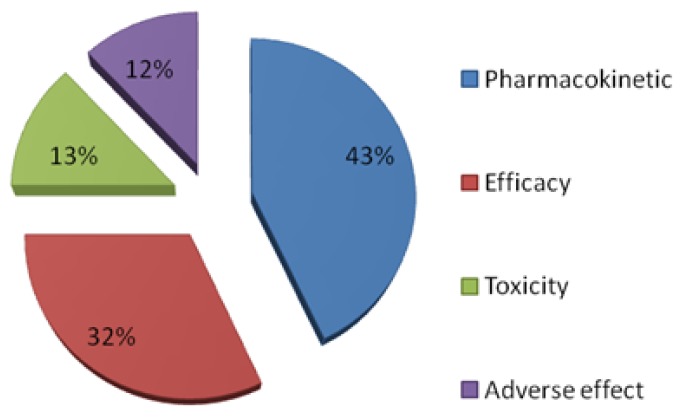
The basic sources of failure in drug development [2].

Many studies on pharmacokinetics report that most of the key causes of costly failures in drug development are because of poor pharmacokinetics and lack of efficacy (Fig. 1). It is therefore essential that these areas be considered as early as possible in the process of drug development [1, 3]. Screening through pharmacokinetic properties and toxicity is usually performed *in vitro* using animal models. These procedures are time-consuming and expensive. Furthermore, the pharmacokinetics of compounds tested on animals may not necessarily be generalized to determine human responses [4, 5].

Considerable research has been done on pharmacokinetic predictions for new drugs and these are performed without any further *in vitro* or *in vivo* experiments. Constructing prediction models involves taking known pharmacokinetic data from a set of drugs already in use that are closely related in terms of their physicochemical properties. Then, the model that is subsequently constructed is used to predict unknown pharmacokinetic parameters of the new entities. Despite recent progress in this field, more research and development is still needed to increase the precision of such predictions.

Quantitative structure-pharmacokinetic relationship (QSPkR) modeling has been successfully used in drug discovery and development processes [6]. These studies use computational tools to determine the correlations between the pharmacokinetic properties and a set of structural descriptors of the molecules in question.

The efficiency of a model for pharmacokinetic prediction depends on the selection of the most appropriate mathematical tools [7]; it also depends on a sufficiently large set of molecular descriptors, and a reliable set of experimental data relating to the purpose of the model. Simple multiple linear regression, often used in earlier QSPkR studies, has been gradually replaced by modern techniques of multivariate analysis such as the artificial neural network (ANN) and genetic algorithms (GA).

A GA is an effective stochastic optimization technique that has been widely employed by chemists for the development of QSPkR and quantitative structure-activity relationship (QSAR) models [8–10]. The GA-QSPR can recognize how the modeled molecular properties are affected by their descriptors. Furthermore, as an optimization technique, GA can work with many descriptors [11].

GAs have often been used in combination with ANNs [12]. Genetic neural networks (GNNs) provide a good method for pruning works that involve large numbers of variables. Comparatively, GNNs have been successfully applied for descriptor selection in QSAR with a fast processing speed [13]. By increasing the ability of computational methods to acquire more descriptors from a molecular structure, GNNs are becoming a more commonly used tool for selecting the most relevant descriptors [14].

Alkaloid drugs were selected for the application in this investigation because they are an important class of drug [15]. Around 1,481 descriptors including zero, one, two, and three-dimensional types, which may influence the pharmacokinetic properties were acquired from Dragon software [16]. A GA was used to select the key molecular descriptors from a wide range of descriptors and ANN was applied to construct QSPkR models.

## Experimental and Methods

### Database of Pharmacokinetic Parameters

Human pharmacokinetic data relating to 39 alkaloid drugs were extracted from different books and literature including: Clarke’s Analysis of Drugs and Poisons [17]; Martindale the Complete Drug Reference [18]; Goodman and Gilman’s the Pharmacological Basic of Therapeutics [19]; Lexi-Comp Program [20]; United States Pharmacopeia [21]; and scientific papers [22–28]. The acquired pharmacokinetic data were normalized within the range of 0–1.

### Structural Descriptors

ChemDraw 8.0 Ultra (CambridgeSoft) was used to generate the molecular structure files from each drug’s generic terms. The files relating to the molecular structure were then imported to Chem3D Ultra (version 8.0; CambridgeSoft) to minimize the energy state of the three-dimensional structure of each molecule by using Molecular Mechanics-2 (MM2). The files generated by Chem3D Ultra were imported to the Dragon program (Version 2.1; Talete srl, Milano, Italy) to generate a total of 1,481 descriptors. Similar to pharmacokinetic data, the acquired descriptor values were normalized to the range of 0–1.

### Outline of Descriptor Selection and Modeling

**Fig. 2:**
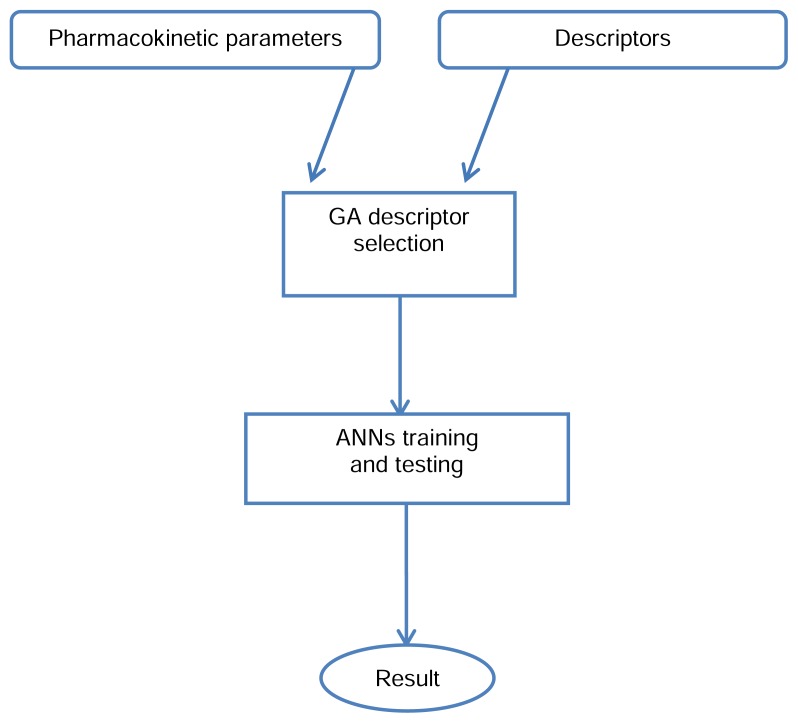
General outline of the QSPkR method

Among the computed descriptors, GA selected a number of zero, one, two, and three-dimensional (1, 2 & 3D) [29] descriptors (Table 1), which had more influence on pharmacokinetic parameters. Outcomes of the GA were then processed by ANN. An overall outline of the processing is shown in Fig. 2.

### GA

**Fig. 3:**
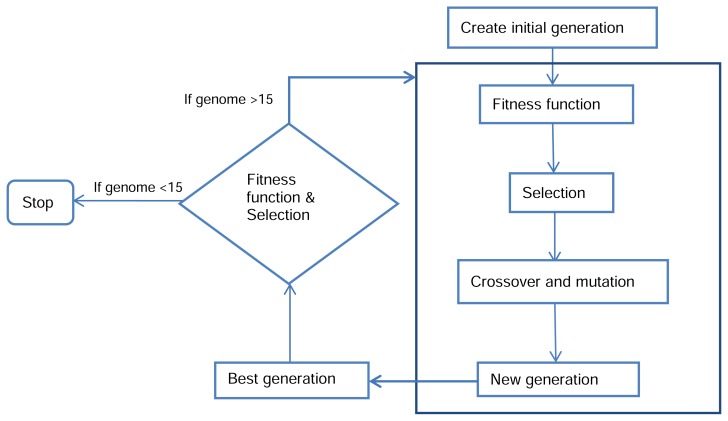
Flowchart describing the steps used in selecting the best subset of descriptors by GA

A GA was developed using Matlab software (MathWorks, version 7.1). GA selects the best subset of descriptors. A flowchart of GA processing is shown in Fig. 3.

### Chromosome and Gene

Each chromosome encodes a different subset of descriptors as binary strings. Each binary value is considered as a gene. “Zero” bit in a chromosome means that the corresponding descriptor is excluded from the subset, whereas “one” means that the corresponding descriptor is included in the subset. The length of each chromosome is equal to the total number of descriptors (1,481 descriptors).

### Production of Initial Chromosome

For the first generation, a selection of 500 chromosomes was randomly generated. Each gene on every chromosome was randomly assigned a value of “zero” or “one”.

### Crossover Function

In the crossover procedure, new chromosomes are generated from a pair of randomly selected parent chromosomes. A crossover probability of one was used in this work. Many methods have been proposed for the crossover technique [31, 32]. Here, two chromosomes were considered as parent chromosomes. A random number was generated between one and 1,481. This number was designated as the location of breaking in the chromosome. Two broken chromosomes were joined to create two new chromosomes.

### Mutation Function

At this stage, a gene in each chromosome was altered with a low probability. The mutating gene was selected randomly. A random number was generated between zero and one and if it was less than 0.1, the mutation was carried out (probability 0.1). In other words, they were altered from zero to one or vice versa. This function could delete or add one descriptor from (to) the descriptor list of chromosomes [31, 32].

### Fitness Function

A fitness function was developed to score the chromosomes and determine the survival probability of the chromosomes. Descriptors with more linear relations with the investigated pharmacokinetic parameter should be identified by the fitness function and grant higher scores. To calculate the overall fitness function of a chromosome, two drugs were randomly selected and the difference between their normalized pharmacokinetic parameters was calculated. Then, the differences between the normalized values of each descriptor (gene) in each pair of drugs were calculated individually and summed to make the total difference of the descriptors. It should be mentioned that only those descriptors that existed in the corresponding chromosome (have one value) were considered in the calculations. An award and penalty parameter with the value of five was considered in the fitness function. If the difference between the normalized pharmacokinetic parameter of each pair of drugs was less than 0.1 (epsilon), the award value was added to the fitness, and the descriptor difference between the two drugs was subtracted from the fitness value [30]. Otherwise, the penalty value was allocated to the fitness value. These calculations were done for all descriptors and for all possible combinations of drug pairs (703 combinations for 39 drugs).

Equ. 1 Fitness function $$\begin{array}{c} {\text{F}}_{\text{i},\text{j},\text{k}}=\{\begin{array}{lll} -\text{Penalty} & \text{if} & \left|{\text{dp}}_{\text{i}}-{\text{dp}}_{\text{j}}\right|\geq \text{epsilon}\\ \text{Award}-\left|{\text{dd}}_{\text{i},\text{k}}-{\text{dd}}_{\text{j},\text{k}}\right| & \text{if} & \left|{\text{dp}}_{\text{i}}-{\text{dp}}_{\text{j}}\right|<\text{epsilon} \end{array}\\ \text{Fitness}=\sum_{\text{descriptor}=1}^{|\text{descriptors}|}\sum_{{\text{d}}_{\text{i}}=1}^{|\text{drugs}|-1}\sum_{{\text{d}}_{\text{j}}={\text{d}}_{\text{i}}+1}^{|\text{drugs}|}{\text{F}}_{{\text{d}}_{\text{i}},{\text{d}}_{\text{j}},\text{descriptor}} \end{array}$$

In Equation 1, dpi is the normalized pharmacokinetic parameter value for ith and ddi, k is the kth normalized descriptor value for the ith drug. As shown in Equation 1, the fitness score will be decreased in cases with a larger number of descriptors. Therefore, as the computation proceeds, the number of genes in the chromosomes decreased for the sake of increasing the fitness score. To prevent the complete abolition of the genes, a limit number of 15 was set for the genes (descriptors) and the computation terminates after this limit is reached.

### Artificial Neural Network

After finding relevant descriptors determined by the GA, the drugs were randomly split into two groups: 33 drugs were allocated to training the ANN and the six remaining drugs were allocated to testing the ANN models (repeated random sub-sampling validation). A back-propagation ANN model was performed using the Matlab neural network toolbox. By performing these operations (from step fitness evaluation to mutation function), the outcomes were continuously surveyed. A three-layered, feed-forward, back-propagation type of network, based on the Levenberg-Marquardt back-propagation algorithm, was used for all models, and it contained a bias neuron in each layer and a single neuron in the output layer. Weight adjustment was performed according to the generalized delta rule [33]. The epoch was set at 50.

In this work, the correlation coefficient model was used to evaluate efficacy. The correlation coefficient was calculated by Equation 2 [34]:

Equ. 2 $$\text{R}=\sqrt{1-\frac{\sum_{\text{i}=1}^{\text{N}}{\left({\text{y}}_{\text{i}}^{\text{obs}}-{\text{y}}_{\text{i}}^{\text{pred}}\right)}^{2}}{\sum_{\text{i}=1}^{\text{N}}{\left({\text{y}}_{\text{i}}^{\text{obs}}-{\bar{\text{y}}}^{\text{obs}}\right)}^{2}}}$$

Where N is the set size, *y_i_* *^obs^* is the observed value for compound i and *y_i_* *^predict^* is the predicted value for compound i.

Another statistical parameter was the root mean square error (RMSE). The RMSE represents deviations of the predicted parameters from the experimental value and is calculated by Equation 3 [35].

Equ. 3 $$\text{RMSE}=\sqrt{\frac{\sum_{\text{i}=1}^{\text{n}}{\left({\text{y}}_{\text{i}}^{\text{obs}}-{\text{y}}_{\text{i}}^{\text{pred}}\right)}^{2}}{\text{n}}}$$

Where *n* is the total number of compounds; *y_i_* *^obs^* is the observed dependent value, and *y_i_* *^predict^* is the predicted dependent value.

Another statistical parameter that has been used was the normalized root mean square error (NRMSE), which was calculated by Equation 4

Equ. 4 $$\text{NRMSE}=\frac{\text{RMSE}}{{\text{x}}_{\text{max}}^{\text{obs}}-{\text{x}}_{\text{min}}^{\text{obs}}}$$

Wherex_max_^obs^ *and* x_min_^obs^ are maximum and minimum values of observed values for each pharmacokinetic parameter, respectively.

## Results and Discussion

GA descriptor selection is fast and flexible. Furthermore, ANN modeling is a strong and expert tool [36] for working with a large number of descriptors. It is known that a larger data set for training ANN models leads to models with better efficiency [37]. However, the collection of pharmacokinetic data for a large number of drugs is limited due to a limited number of drugs and a lack of adequate and reliable scientific sources [38, 39]. There are an inadequate number of alkaloid drugs cited in the related literature, so extracting pharmacokinetic parameters is problematic. These conditions made it reasonable to choose a combined technique of GA-ANN for use in the study. By only using GA, the models may not have been valid when based on a training set of less than 1000 compounds [40–42]. Besides, the models based on only ANN have the problem of over fitting when there are a large number of descriptors [43]. Thus, GA was applied to make selections for descriptors that are more relevant, and ANN was applied to make models of predictions. This combination could be used to compensate for the specific disadvantages of each method [44].

### Homogeneity

Results of the independent sample t-test showed no significant difference between the training and test sets for all examined pharmacokinetic parameters. The Levene test for homogeneity variance revealed no significant difference (Table 2).

Alkaloids are characterized by substantial structural diversity and there is almost no unique classification for this group [36–40]. Due to this diversity, there is a diverse range of pharmacokinetic parameters in this group [45].

### Descriptor Analysis

The most relevant descriptors for each pharmacokinetic parameter chosen by GA are listed in Tables 3–5.

These descriptors were previously reported to have significance rule in modeling pharmacokinetic parameters [46]. For each pharmacokinetic parameter prediction, at least half of the effective descriptors were of the 3D type. Previous studies have also reported on the key role of 3D descriptors in the predictions of pharmacokinetic parameters such as plasma protein binding [47, 48]. 3D conformation of biological molecules and drug molecules can explain the important role of these types of descriptors in pharmacokinetic predictions [49, 50].

Topological descriptors, which are calculated from the 2D structure of molecules, are single-valued descriptors [51]. They are sensitive to size, shape, and branching [52, 53]. Systemic clearance is extensively affected by topological descriptors. The same result has been reported for other drugs [54].

### Optimum Models

**Fig. 4:**
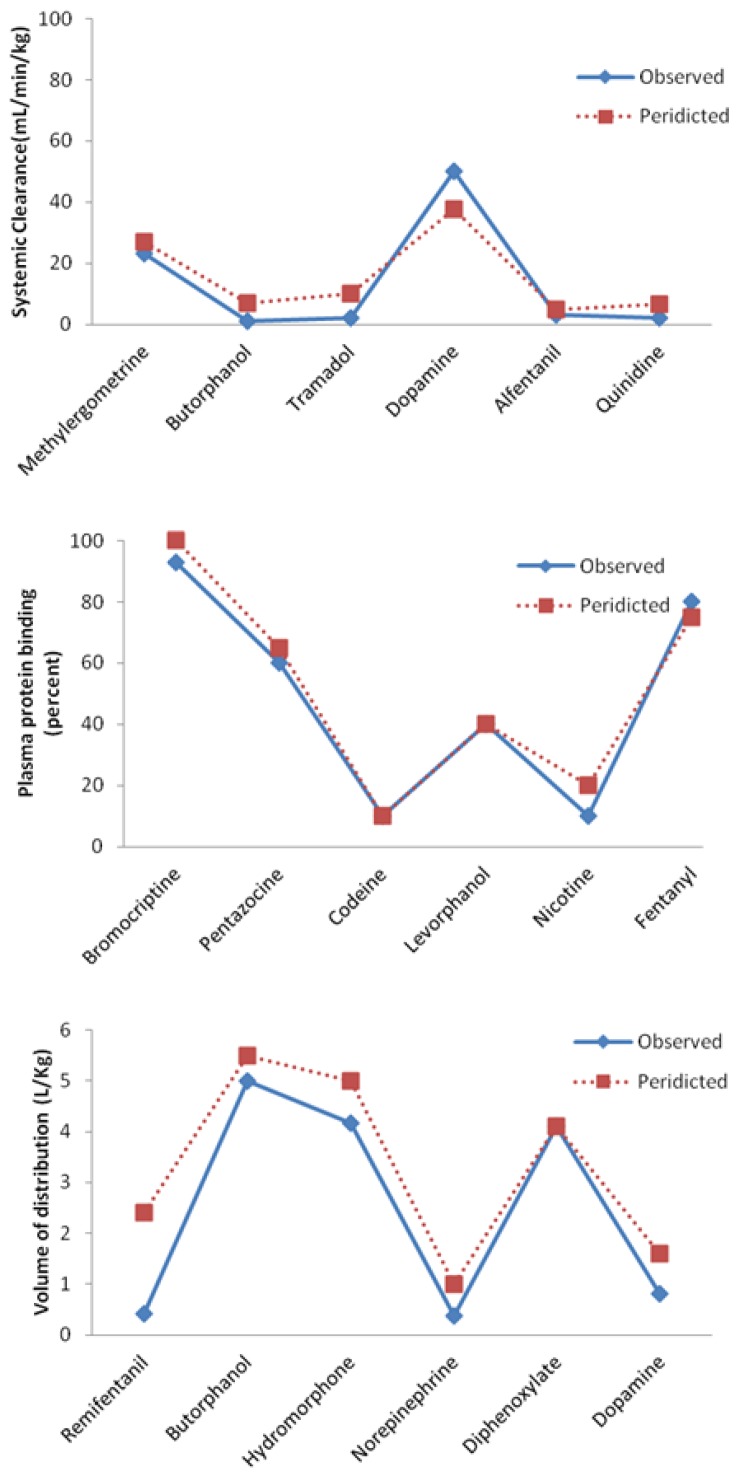
Predicted vs. observed experimental pharmacokinetic values for optimum ANN models

The predicted and observed values for each drug in a test set were compared and results are represented in Fig. 4 and Table 6.

Values determined by the correlation coefficient for the constructed models ranged from 0.957 to 0.991 (Table 6). Correlation coefficient reports in a number of other studies revealed a wide range of values in different studies: 0.855 to 0.992 for predictions of pharmacokinetic parameters [44–56], 0.71 to 0.79 for predictions of oral bioavailability of diverse compounds [57], 0.78 for human oral bioavailability, 0.92 for plasma protein binding, 0.81 for urinary excretion [58], and 0.85 for blood-brain barrier penetration [35]. The correlation coefficient values of the current work were acceptable despite the limited number of drugs that were studied. This could be explained by the large number of molecular descriptors that were generated by the Dragon program, emphasizing the known fact that using more descriptors will improve the accuracy of the GA descriptor selection and ANN modeling [11, 59].

The NRMSE of every test set provides an overall view of the prediction ability of a model. Values for NRMSE were different for each model and indicate differences in terms of the ability of ANN to make predictions for each parameter [60].

Clearance is an extremely important parameter for clinical application [61]. It is also useful for studying the elimination mechanism [62]. Systemic clearance is the total clearances of individual organs, and is very complex [63]. Drugs undergo different and stepwise metabolic pathways such as Phase I, Phase II, and conjugation transformations [64, 65]. However, the same as for other investigated pharmacokinetic parameters, the systemic clearance model has acceptable RMSE and NRMSE values (Table 6). Furthermore, a good agreement was observed between the predicted values of volume of distribution and plasma protein binding and hence, small RMSE and NRMSE were seen. Turner *et al*. reported that RMSE values for plasma protein binding were dependent on the extent of drug protein binding. They reported a higher RMSE for drugs with lower protein binding and a lower RMSE for drugs with higher protein binding. Note that drugs with high plasma protein binding are important in clinical practice. Compared to reports of Turner *et al*., the efficiency of predictions for plasma protein binding were improved by the technique applied in this study due to the use of GA and the inclusion of 3D descriptors in predictions [47].

## Conclusion

GA and the modeling performed by ANN were effective in choosing the most relevant descriptors for each parameter. The prediction efficiency of the developed models was acceptable for the investigated pharmacokinetic parameters. Using a large number of descriptors, especially the 3D type, could explain the enhanced efficiency of selecting predictions that has been determined in this work.

It is expected that more data will be available for testing alkaloid drugs in future literature, and this may lead to further improvements in future QSPkR models.

## Figures and Tables

**Tab. 1:** List of molecular descriptors

Descriptor type	List of descriptor groups
0 Dimensional	Constitutional descriptor
1 Dimensional	Functional groups Atom-centered fragments Empirical descriptors Properties
2 Dimensional	Topological descriptors Molecular walk counts BCUT descriptors Galvez topol. Charge indices 2D autocorrelations
3 Dimensional	Charge descriptors Aromaticity indices Geometrical RDF descriptors Randic molecular descriptors 3D-MoRSE descriptors WHIM descriptors GETAWAY descriptors

**Tab. 2:** Statistical analysis

Models	Levene^a^	T-test^b^
Systemic clearance	0.401	0.679
Volume of distribution	0.309	0.517
Plasma protein binding	0.644	0.330

a Levene homogeneity of variance.

b Independent samples T-test.

**Tab. 3:** The most relevant descriptors for systemic clearance

Descriptor type	Symbol and meaning
Topological	VEp1: eigenvector coefficient sum from polarizability weighted distance metrix
BCUT	BEHv3: highest eigenvalue n.3 of burden matrix/weighted by atomic van der waals volumes
Galves topological charge indexes	GGI5: topological charge index of order5
2D atocorrelatione	ATS1m: Broto-Moreau autocorrelation of a topological structure – lag1/weighted by atomic masses. ATS2e: Broto-Moreau autocorrelation of a topological structure –lag2/weighted by atomic Sanderson electro negativities. ATS5p: Broto-Moreau autocorrelation of a topological structure –lag5/weighted by atomic polarizabilities. GATS5e: Garry autocorrelation – lag5/weighted by atomic Sandeson electro negativities.
Geometrical	SPAN: span R. G(N…N): sum of geometrical distance between N…N
RDF	RDF120m: Radial Distribution Function – 12.0/weighted by atomic masses. RDF150v: Radial Distribution Function – 15.0/weighted by atomic van der Waals volumes.
3D- MoRSE	Mor11u: 3D-MoRSE – signal11/unweighted. Mor20v: 3D-MoRSE – signal20/weighted by atomic van der Waals volumes. Mor26v: 3D-MoRSE – signal26/weighted by atomic van der Waals volumes.

**Tab. 4:** The most relevant descriptors for volume of distribution

Descriptor type	Symbol and meaning
BCUT	BEHe2: highest eigenvalue n.2 of Burden matrix/weighted by atomic Sanderson electro negativities.
Galves topological charge indexes	JGI2: mean topological charge index of order2.
2D atocorrelatione	MATS7m: mean autocorrelation – lag7/weighted by atomic masses. MATS8p: mean autocorrelation – lag8/weighted by atomic polarizabilities. GATS4e: Geary autocorrelation – lag4/weighted by atomic Sanderson electro negativities.
Geometrical	SPAM: average span R.
RDF	RDF155m: Radial distribution function- 15.5/weighted by atomic masses.
3D- MoRSE	Mor30u: 3D- MoRSE – signal 30/unweighted. Mor30m: 3D- MoRSE – signal 30/weighted by atomic masses. Mor04p: 3D- MoRSE – signal 04/weighted by atomic polarizabilities.
WHIM	P1e: 1^st^ component shape directional WHIM index/weighted by atomic Sanderson electro negativities. L2p: 2^nd^ component size directional WHIM index/weighted by atomic polarizabilities.
GETAWAY	HATS2m: leverage-weighted autocorrelation of lag2/weighted by atomic masses. H7e: H autocorrelation of lag7/weighted by atomic Sanderson electro negativities.

**Tab. 5:** The most relevant descriptors for plasma protein binding

Descriptor type	Symbol and meaning
Galves topological charge indexes	JGI10: mean topological charge index of order10.
2D atocorrelatione	ATS1v: broto – oreau autocorrelation of a topological structure – lag1/weighted by atomic van der Waals volumes. MATS1v: Moran autocorrelation – lag1/weighted by atomic van der waals volumes. MATS2e: Moran autocorrelation – lag2/weighted by atomic Sanderson electro negativities. MATS6p: Moran autocorrelation – lag6/weighted by atomic polarizabilities. GATS1m: geary autocorrelation – lag 1/weighted by atomic masses.
3D- MoRSE	Mor29u: 3D- MoRSE – signal 29/unweighted. Mor24v: 3D- MoRSE – signal 24/weighted by atomic van der Waals volumes. Mor22e: 3D- MoRSE – signal 22/weighted by atomic Sanderson electro negativities.
WHIM	E3p: 3^rd^ component accessibility directional WHIM index/weighted by atomic polarizabilities.
GETAWAY	H7m: H autocorrelation of lag7/weighted by atomic masses. H5p: H autocorrelation of lag5/weighted by atomic polarizability. R3u+: R maximal autocorrelation of lag3/unweighted. R8m: R autocorrelation of lag8/weighted by atomic masses.

**Tab. 6:** Correlation coefficient, RMSE, and NRMSE values for each model

	R^a^	RMSE^b^	NRMSE^c^
Systemic clearance (mL/min/Kg)	0.972	7.03	0.151
Volume of distribution (L/Kg)	0.957	0.995	0.263
Plasma protein binding (%)	0.991	0.055	0.423

a Correlation coefficient;

b Root mean square error;

c Normalized RMSE
